# Quantifying the Effects of Climate Factors on Carbon Monoxide Poisoning: A Retrospective Study in Taiwan

**DOI:** 10.3389/fpubh.2021.718846

**Published:** 2021-10-14

**Authors:** Chien-Ho Wang, Shih-Chieh Shao, Kai-Cheng Chang, Ming-Jui Hung, Chen-Chang Yang, Shu-Chen Liao

**Affiliations:** ^1^Department of Emergency Medicine, Keelung Chang Gung Memorial Hospital, Keelung, Taiwan; ^2^Department of Pharmacy, Keelung Chang Gung Memorial Hospital, Keelung, Taiwan; ^3^Department of Pharmacy, Linkou Chang Gung Memorial Hospital, Taoyuan, Taiwan; ^4^Section of Cardiology, Department of Internal Medicine, Keelung Chang Gung Memorial Hospital, Keelung, Taiwan; ^5^Community Medicine Research Center, Keelung Chang Gung Memorial Hospital, Keelung, Taiwan; ^6^College of Medicine, Chang Gung University, Taoyuan, Taiwan; ^7^Institute of Environmental and Occupational Health Sciences, School of Medicine, National Yang Ming Chiao Tung University, Taipei, Taiwan; ^8^Division of Clinical Toxicology & Occupational Medicine, Department of Medicine, Taipei Veterans General Hospital, Taipei, Taiwan

**Keywords:** carbon monoxide poisoning, accidental poisoning, weather warnings, weather parameters, climate factors, climate effects on CO poisoning

## Abstract

**Background:** Carbon monoxide (CO) poisoning is the leading cause of poisoning death worldwide, but associations between CO poisoning and weather remain unclear.

**Objective:** To quantify the influence of climate parameters (e.g., temperature, relative humidity, and wind speed) on the incidence risk of acute CO poisoning in Taiwan.

**Methods:** We used negative binomial mixed models (NBMMs) to evaluate the influence of weather parameters on the incidence risk of acute CO poisoning. Subgroup analyses were conducted, based on the seasonality and the intentionality of acute CO poisoning cases.

**Results:** We identified a total of 622 patients (mean age: 32.9 years old; female: 51%) with acute CO poisoning in the study hospital. Carbon monoxide poisoning was associated with temperature (beta: −0.0973, rate ratio (RR): 0.9073, *p* < 0.0001) but not with relative humidity (beta: 0.1290, RR: 1.1377, *p* = 0.0513) or wind speed (beta: −0.4195, RR: 0.6574, *p* = 0.0806). In the subgroup analyses, temperature was associated with the incidence of intentional CO poisoning (beta: 0.1076, RR: 1.1136, *p* = 0.0333) in spring and unintentional CO poisoning (beta: −0.1865, RR: 0.8299, *p* = 0.0184) in winter.

**Conclusion:** Changes in temperature affect the incidence risk for acute CO poisoning, but the impact varies with different seasons and intentionality in Taiwan. Our findings quantify the effects of climate factors and provide fundamental evidence for healthcare providers to develop preventative strategies to reduce acute CO poisoning events.

## Introduction

Carbon monoxide (CO) poisoning is a global issue of great significance for public health and societal costs (1–4). According to worldwide epidemiological data, in 2017 the cumulative incidence and mortality rates of CO poisoning were about 137 cases and 4.6 deaths per one million person-years, respectively. Over the past 25 years, the worldwide incidence has remained stable, but the mortality rate has declined by 40% due to continued public health education and the improvement of treatment (5, 6). However, epidemiological features vary in different countries. According to statistics from Taiwan's government, and contrary to the global trend, the mortality rate of unintentional poisoning continued to increase from 1.6 to 3.5 per million person-years during the period of 1997–2003 in Taiwan (7, 8), which was mostly related to old piping and improper use of heating systems. Moreover, the incidence of intentional CO poisoning (e.g., charcoal-burning suicide) also surged from 0.22 to 9.8 per million person-years during 1999–2019 (9, 10), and this has now became the second most common cause of intentional deaths (11).

Identifying risk factors is vital to developing appropriate precautions and weather-related factors have been considered one of the most important risk factors for acute CO poisoning. A previous study by Tiekuan Du et al. in Beijing indicated that temperature was inversely correlated with the incidence of acute CO poisoning (*r* = −0.467) (12). Another study from Roca-Barceló Aina et al. in the UK also demonstrated clear seasonality in the incidence of acute CO poisoning (13). However, the risk factors may vary depending on the intention behind the CO poisoning; for example, unintentional CO poisonings probably related to improper installation of household heating systems (14) were associated with seasonal variations or particular weather conditions, such as colder seasons or sudden changes in temperature (15–17). Some specific demographics with high risk of intentional CO poisoning were identified as male patients (6), patients with psychiatric disorders (e.g., depression or schizophrenia) (18) and patients of unmarried status (8, 9, 19, 20). Thus, the study of CO poisoning cannot merely involve research of one specific region or any single risk factor.

However, current evidence regarding the risk factors for acute CO poisoning, especially through weather impacts has mostly come from Western countries (21, 22). The generalizability into Asian countries with different cultural and climatic patterns remains unclear. For example, the climate of Taiwan is warm (mean annual temperature: 24.6°C), rainy (mean rainy days: 146 days) and humid (mean relative humidity: 79.43%), so the weather impacts on CO poisoning in Taiwan may differ from previous reports. In addition, previous studies have mostly explored the association between temperature changes and CO poisoning, while the influence of other important weather factors (e.g., relative humidity and wind speed) on this issue remains unclear (12, 21). We therefore conducted this study to fill the knowledge gap and assist healthcare providers and policy makers to develop more strategies based on weather parameters to avoid intentional and unintentional CO poisoning.

## Materials and Methods

### Study Design and Setting

We conducted a retrospective study by analyzing the electronic medical records of Linkou Chang Gung Memorial Hospital in Taiwan from 2010 to 2015. Linkou Chang Gung Memorial Hospital is the largest medical center in Taiwan, handling over 8,000 admissions monthly, and 17,000 and 330,000 patient visits for emergency and outpatient care, respectively. This study was approved by the Chang Gung Medical Foundation Institutional Review Board (IRB: CMRPG2E0091). Given the retrospective nature of this study, the need for informed consent was waived by the above mentioned committee.

### Selection of Study Patients

We included adult patients diagnosed with acute CO poisoning, defined by carboxyhemoglobin (COHb) levels >5% in non-smokers and >10% in smokers (23), who were admitted or transferred to the emergency department of the study hospital. The COHb levels were measured by arterial blood gas at the initial medical institution or the Linkou Chang Gung Memorial Hospital. We excluded patients without complete information (e.g., incomplete documentation of medical records or transfer sheets).

### Data Collection

We collected patients' demographics, including sex, age, past history of psychiatric diseases, marital status, coma scale upon arrival at the study hospital, sources of CO exposure, and intentional or unintentional purpose. This was performed by two clinical physicians (CHW and SCL) manually reviewing the electronic medical records or the medical transfer sheets. We obtained the mean values of daily weather parameters (e.g., temperature, relative humidity, and wind speed) during the study period from the Linkou weather station of the Central Weather Bureau of Taiwan, the official meteorological research and forecasting institution in Taiwan.

### Outcomes

The primary outcomes were the effects from changes in daily mean temperature, relative humidity, and wind speed on the incidence of acute CO poisoning. We calculated the incidence of acute CO poisoning as the daily number of acute CO poisoning cases divided by the daily number of all patients seeking medical services in the emergency settings of the study hospital.

### Statistical Analysis

We compared baseline demographic characteristics in acute CO poisoning cases based on their intentionality (intentional or unintentional) using independent *t*-tests for continuous variables and Chi-square tests for categorical variables. We used negative binomial mixed models (NBMMs) to deal with the possible issue of over-dispersion while estimating the association between CO poisoning cases and environmental factors for correlated count data. Specifically, the environmental record of the year (i.e., 2010–2015) is included as a random factor and weather parameters are considered as fixed effects in the mixed model. To determine if the effects of weather parameters on the incidence of acute CO poisoning cases varied between intentional and unintentional poisonings and different seasons (i.e., spring, summer, fall, and winter), we also performed subgroup analyses based on intentionality and seasonality. We calculated the rate ratio (RR) of each weather parameter for the incidence of acute CO poisoning based on the multi-variable NBMMs. Moreover, we determined the variance inflation factor (VIF) to test for multicollinearity, and all VIF-values were <2, indicating low collinearity among weather parameters. All *P*-values were two-sided and those <0.05 were considered statistically significant. We performed analyses using SAS Enterprise statistical software (version 5.1).

## Results

### Demographic Characteristics of the CO Poisonings

**Figure 1:**
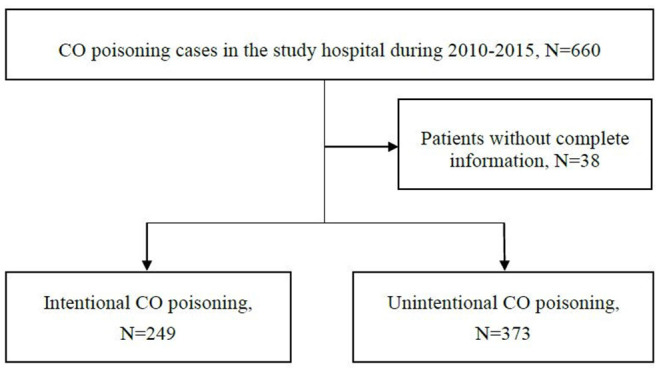
Study flow chart.

During the period 2010–2015, we initially identified a total of 660 acute CO poisoning cases in the emergency settings in the study hospital. After excluding 38 cases due to incomplete data (Figure 1), we finally included in this study 622 CO poisoning cases with a mean age of 32.9 years old; 305 (49.0%) were male. We found 249 (40.0%) and 373 (60.0%) cases with intentional and non-intentional CO poisoning, respectively. The greatest differences between the intentional and the non-intentional CO poisoning groups were found in the source of CO poisoning (charcoal burning: 92.3 vs. 5.6%, *p* < 0.05; water heater incomplete combustion or incorrect use: 1.6 vs. 87.9%, *p* < 0.05) and the history of psychiatric diseases (42.6 vs. 1.6%, *p* < 0.05). Detailed comparisons of baseline characteristics in CO poisoning cases with different intentionality are listed in Table 1.

**Table 1:** Demographics and clinical data of CO poisoning cases.

**Variable**	**Intentional (*N* = 249)**	**Unintentional (*N* = 373)**	***P*-values**
Male, *n* (%)	146 (58.6%)	159 (42.6%)	<0.05
Age, mean (SD) years old	36.4 (12.3)	30.6 (18.4)	<0.05
History of psychiatric diseases, *n* (%)	106 (42.6%)	6 (1.6%)	<0.05
GCS <9, *n* (%)	64 (25.7%)	8 (2.1%)	<0.05
Married status, *n* (%)	72 (28.9%)	154 (41.2%)	0.002
Season of the CO poisoning, *n* (%)			
Spring (March–May)	59 (23.7%)	124 (33.2%)	<0.05
Summer (June–August)	62 (24.9%)	12 (3.2%)	<0.05
Fall (September–November)	63 (25.3%)	30 (8.0%)	<0.05
Winter (December–February)	65 (26.1%)	207 (55.4%)	<0.05
Transferred from outside institutions, *n* (%)	133 (53.4%)	217 (58.1%)	0.241
Source of CO poisoning, *n* (%)			
Charcoal burning	230 (92.3%)	21 (5.6%)	<0.05
Water heater incomplete combustion or incorrect use of furnace	4 (1.6%)	328 (87.9%)	<0.05
Other^*^	15 (6.0%)	24 (6.4%)	0.499
Time of arrival at emergency room, *n* (%)			
Day shift (08:01–16:00)	73 (29.3%)	73 (19.6%)	0.004
Evening shift (16:01–00:00)	103 (41.3%)	125 (33.5%)	0.004
Night shift (00:01–08:00)	73 (29.3%)	175 (46.9%)	0.004

*GCS, Glasgow Coma Scale*.

* *Other sources including occupational accident, fire or automobile exhaust*.

### Effects of Weather Changes on the Incidence of Acute CO Poisoning

Overall, the NBMMs indicated that the temperature was significantly associated with the incidence of acute CO poisoning (beta: −0.0973, RR: 0.9073, *p* < 0.0001), and there were no significant associations between the relative humidity (beta: 0.1290, RR: 1.1377, *p* = 0.0513) or wind speed (beta: −0.4195, RR: 0.6574, *p* = 0.0806) and the incidence of acute CO poisoning (Table 2). However, the impact of weather changes on the incidence of acute CO poisoning varied, depending on whether the poisoning was intentional (Table 3) or unintentional (Table 4). No specific weather parameters associated with intentional CO poisoning were observed, but temperature (beta: −0.1945, RR: 0.8233, *p* < 0.0001) and relative humidity (beta: 0.2308, RR: 1.2596, *p* = 0.0379) were significantly associated with the incidence of unintentional CO poisoning. After the subgroup analyses according to different intentionality and seasons, temperature was associated with the incidence of intentional CO poisoning in spring (beta: 0.1076, RR: 1.1136, *p* = 0.0333) and unintentional CO poisoning during winter (beta: −0.1865, RR: 0.8299, *p* = 0.0184).

**Table 2:** The impacts from the changes of weather parameters on the incidence of all CO poisoning.

	**Beta value**	**Standard error**	***P*-value**	Rate ratio^*^
**All seasons (*****N*** **=** **622)**				
Wind speed	−0.4195	0.2400	0.0806	0.6574
Temperature	**−0.0973**	**0.0134**	** <0.0001**	**0.9073**
Relative humidity	0.1290	0.0066	0.0513	1.1377
**Spring (*****N*** **=** **183)**				
Wind speed	−0.3861	0.4773	0.4188	0.6797
Temperature	0.0624	0.0383	0.1035	1.0644
Relative humidity	0.0129	0.0111	0.2461	1.0129
**Summer (*****N*** **=** **74)**				
Wind speed	−0.4786	0.5390	0.3749	0.6197
Temperature	−0.0223	0.0445	0.6159	0.9779
Relative humidity	0.0363	0.0185	0.0501	1.0370
**Fall (*****N*** **=** **93)**				
Wind speed	0.1178	0.3378	0.7275	1.1250
Temperature	−0.0960	0.0767	0.2112	0.9085
Relative humidity	0.0001	0.0154	0.9935	1.0001
**Winter (*****N*** **=** **272)**				
Wind speed	−1.1504	0.6848	0.0936	0.3165
Temperature	−0.0785	0.0423	0.0644	1.0816
Relative humidity	0.0208	0.0147	0.1567	1.0210

* *Rate ratio is calculated by exp (beta value). Bold values are statistically significant (p-value < 0.05)*.

**Table 3:** Subgroup analyses of the changes of weather parameters on the incidence of intentional CO poisoning.

	**Beta value**	**Standard error**	***P*-value**	Rate ratio^*^
**All seasons (*****N*** **=** **249)**				
Wind speed	−0.3607	0.2420	0.1364	0.6972
Temperature	0.0084	0.0135	0.5311	1.0085
Relative humidity	0.0030	0.0072	0.6749	1.0030
**Spring (*****N*** **=** **59)**				
Wind speed	−0.9221	0.7229	0.2027	0.3977
Temperature	**0.1076**	**0.0504**	**0.0333**	**1.1136**
Relative humidity	−0.0202	0.0152	0.1829	0.9800
**Summer (*****N*** **=** **62)**				
Wind speed	0.0079	0.4236	0.9851	1.0079
Temperature	0.0135	0.0413	0.7440	1.0136
Relative humidity	0.0269	0.0170	0.1141	1.0272
**Fall (*****N*** **=** **63)**				
Wind speed	0.3203	0.3312	0.3339	1.3775
Temperature	−0.1192	0.0802	0.1377	0.8876
Relative humidity	0.0073	0.0156	0.6404	1.0073
**Winter (*****N*** **=** **65)**				
Wind speed	−0.6555	0.5510	0.2346	0.5192
Temperature	0.0245	0.0360	0.4964	1.0248
Relative humidity	0.0127	0.0133	0.3389	1.0128

* *Rate ratio is calculated by exp (beta value). Bold values are statistically significant (p-value < 0.05)*.

**Table 4:** Subgroup analyses of the changes of weather parameters on the incidence of unintentional CO poisoning.

	**Beta value**	**Standard error**	***P*-value**	Rate ratio^*^
**All seasons (*****N*** **=** **373)**				
Wind speed	−0.7640	0.4572	0.0948	0.4658
Temperature	**−0.1945**	**0.2481**	** <0.0001**	**0.8233**
Relative humidity	**0.2308**	**0.0111**	**0.0379**	**1.2596**
**Spring (*****N*** **=** **124)**				
Wind speed	−0.3874	0.6495	0.5511	0.6788
Temperature	0.0765	0.0510	0.1346	1.0795
Relative humidity	0.0277	0.0153	0.0710	1.0281
**Summer (*****N*** **=** **12)**				
Wind speed	−1.8771	1.2342	0.1289	0.1530
Temperature	−0.0845	0.1040	0.4166	0.9189
Relative humidity	0.0363	0.0339	0.2845	1.0370
**Fall (*****N*** **=** **30)**				
Wind speed	−2.2466	1.6938	0.1853	0.1058
Temperature	0.0871	0.2615	0.7392	1.0910
Relative humidity	−0.0457	0.0543	0.4003	0.9553
**Winter (*****N*** **=** **207)**				
Wind speed	−1.3471	1.2667	0.2881	0.2600
Temperature	**−0.1865**	**0.0789**	**0.0184**	**0.8299**
Relative humidity	0.0409	0.0257	0.1123	1.0417

* *Rate ratio is calculated by exp (beta value). Bold values are statistically significant (p-value < 0.05)*.

## Discussion

Development of a local body of data regarding risk factors for CO poisoning has been crucial for reducing the incidence of CO poisoning. This study provides local data on CO poisoning in an Asian setting and demonstrates not only that weather changes could affect the incidence of acute CO poisoning, but also that climate impacts on CO poisoning vary, depending on the intentionality of the poisonings. We have quantified the relative effects of weather changes on CO poisoning, and our findings indicate that increased risks of unintentional CO poisoning are associated with lower temperature and higher relative humidity. However, no significant impacts of weather factors on intentional CO poisoning were observed in our study.

### Temperature Changes and CO Poisoning

Several studies have illustrated that the incidence of CO poisoning varies with different seasons (13, 16, 24) and temperature changes are the leading risk factors. A previous study by Shie HG et al. in Taiwan found that while the daily maximum temperature was lower than 18.4°C, the odds ratio for unintentional CO poisoning incidence was increased 2.15 times, compared to the odds ratio when the temperature exceeded 27.1°C (8). Similarly, temperature changes were considered to be associated with the incidence of suicide attempts (25, 26). However, in a recent study the correlation between temperature changes and suicide incidence remained inconclusive, probably due to different regions or study designs (27). In our study, we not only found that lower temperature leads to higher risk of CO poisoning, but also that an increase of 1°C may lead to a 10% decrease in acute CO poisoning incidence. The higher incidence of unintentional CO poisoning at lower temperatures may be the result of people closing doors and windows to keep warm at home, thus preventing air circulation and increasing indoor CO levels, especially with improper installation of household heating systems. This tendency of linear association between temperature and CO poisoning events was also observed in other studies (12, 17), and may reflect the increasing probability of people staying indoors and using household heating systems as the weather gets colder.

### Relative Humidity Changes and CO Poisoning

Relative humidity reflects the moisture content of the atmosphere at a given temperature. Based on relevant studies in Taiwan, relative humidity has a significant impact on apparent temperature, especially in subtropical regions. Higher relative humidity in cold weather makes people feel it is colder than the actual temperature (28), which may in turn lead to increased heater usage. One study in Romania also revealed direct association between humidity and the CO concentration in the environment (29). However, studies by Du et al. and Ruan et al. have indicated that relative humidity in the domicile did not significantly affect the incidence of acute CO poisoning (12, 17). This inconsistency may stem from the fact that some previous studies failed to conduct subgroup analyses to evaluate relative humidity and CO poisoning with different intentions. In this study, our findings support that higher relative humidity is only associated with an increased risk of unintentional CO poisoning, but not of intentional CO poisoning.

### Wind Speed Changes and CO Poisoning

Carbon monoxide tends to concentrate near its sources if wind speed is low, especially when it is under 1.3m/s (30). Hung et al. have indicated that higher wind speed and higher temperature were the most important climate factors to prevent acute CO poisoning occurrences (17). In our study, however, there was no correlation between wind speed and CO poisoning. This result implies that in CO poisoning events mostly attributed to indoor heating systems or charcoal burning, the outdoor wind speed had little effect on the indoor CO concentration if the indoor environment was poorly ventilated.

### Seasonality and CO Poisoning

Seasonal variation in cases of CO poisoning was observed in one previous study in Korea (31). In our study, over 73% of CO poisoning cases happened during spring and winter with four times more CO poisoning cases in winter compared to summer. We also found correlation between temperature and intentional poisoning in spring and unintentional poisoning in winter after conducting subgroup analyses. The higher incidence of unintentional CO poisoning in winter is closely related to elevated indoor CO concentration from improper installation and use of heating systems.

In Taiwan, April and May are the rainy seasons; the weather is humid, hot, and unstable. Some studies have illustrated a significant seasonal variation in suicide attempts with a distinct seasonal pattern, especially in spring and autumn, found in females (32–34). Other researchers have documented a positive association between climate factors like the intensity of sunlight (longer day length), higher temperature, more humidity, and suicide (35, 36). As we know, serotonin concentration and tryptophan (the primary precursor of serotonin) levels in the brain are often associated with impulsive control and aggressive behaviors; both neurotransmitters show a prominent seasonal rhythm with lower plasma levels measured in spring in comparison to other seasons (37). These reasons may partly explain the increase in intentional CO poisoning in the spring, as the temperature rises, observed in our study.

### Our Findings in Public Health

Consistent with previous studies from Western countries, our study confirms the influence of temperature on CO poisoning in Taiwan (8, 16). Our study also recognizes that relative humidity is a further potential risk factor for unintentional CO poisoning, which has never been evaluated in previous studies. Our findings may be clinically important to build up a forecasting system for CO poisoning occurrences by incorporating weather parameters in the future. For example, when weather forecasts predict higher humidity and lower temperature approaching, we may issue weather warnings to remind the public to be aware of increased risks of CO poisoning and to take related measures (e.g., opening windows, being cautious with heating systems) in advance.

### Strengths and Limitations

To the best of our knowledge, this is the first study reporting quantitative risk evaluations of several weather parameters on CO poisoning incidence in Taiwan. Most importantly, we conducted subgroup analyses based on the intentionality of CO poisoning, and found the effects of weather changes on CO poisoning differed, depending on the intention behind the poisoning. However, our findings should be interpreted cautiously due to the nature of retrospective studies. First, we did not evaluate other risk factors of CO poisoning, since we only focused on weather changes in this study. Second, we only used the daily mean values of weather parameters to evaluate the risks of CO poisoning, but weather parameters undergo huge variations in Taiwan. Third, cigarette smoking was a special issue, since smokers often have higher COHb levels. However, it was not always feasible to identify smokers in our routine clinical practice. In our study, 21% (131/622) of the patients were identified as smokers, but for the majority of the patients uncertainty about their smoking status remained, which hindered further analysis. Fourth, although this study was drawn from the largest medical center in Taiwan, climate effects on CO poisoning are regionally highly variable. The generalizability of the result is subject to regional considerations and should be further confirmed in other medical institutions. Finally, this city-based study depicted the situation of CO intoxication in northern Taiwan. The results should be interpreted with caution if applying to individuals.

## Conclusions

Changes in daily mean temperature and relative humidity affect the incidence of unintentional CO poisoning, but not of intentional CO poisoning in Taiwan. Our findings quantified the effects of climate factors and provided fundamental evidence for healthcare providers and policy makers to develop preventative strategies based on weather changes and patient's intentions to reduce acute CO poisoning events.

## Data Availability Statement

The original contributions presented in the study are included in the article/supplementary material, further inquiries can be directed to the corresponding author/s.

## Author Contributions

S-CL and S-CS: conceptualization, review, and editing. S-CL, S-CS, K-CC, and C-HW: methodology. S-CS, K-CC, and C-HW: data curation, investigation, and formal analysis. C-HW: original draft and visualization. S-CL: funding acquisition. M-JH, C-CY, and S-CL: supervision. All authors have read and agreed to the published version of the manuscript.

## Funding

The work was funded by Chang Gung Medical Foundation (grant number: CMRPG2E0091).

## Conflict of Interest

The authors declare that the research was conducted in the absence of any commercial or financial relationships that could be construed as a potential conflict of interest.

## Publisher's Note

All claims expressed in this article are solely those of the authors and do not necessarily represent those of their affiliated organizations, or those of the publisher, the editors and the reviewers. Any product that may be evaluated in this article, or claim that may be made by its manufacturer, is not guaranteed or endorsed by the publisher.
